# High expression of FUT3 is linked to poor prognosis in clear cell renal cell carcinoma

**DOI:** 10.18632/oncotarget.17717

**Published:** 2017-05-09

**Authors:** Li Meng, Le Xu, Yuanfeng Yang, Lin Zhou, Yuan Chang, Tianming Shi, Cheng Tan, Huimin An, Yu Zhu, Jiejie Xu

**Affiliations:** ^1^ Department of Urology, Ruijin Hospital, Shanghai Jiao Tong University School of Medicine, Shanghai, China; ^2^ Department of Urology, Zhongshan Hospital, Fudan University, Shanghai, China; ^3^ Department of Biochemistry and Molecular Biology, School of Basic Medical Sciences, Fudan University, Shanghai, China

**Keywords:** clear cell renal cell carcinoma, fucosyltransferase-III, prognosis, overall survival, recurrence free survival

## Abstract

**Background and Purpose:**

Some of the fucosylation catalyzed by fucosyltransferase-III mediates the epithelial-mesenchymal transition and enhances tumor cell-macrophage signaling, which promotes malignant transforming and immune evasion. The aim of the study was to investigate the association between the expression of fucosyltransferase-III and clinical outcomes of patients with clear-cell renal cell carcinoma after surgery.

**Results:**

High fucosyltransferase-III expression was associated with a greater risk of recurrence (*p* = 0.002) and shortened overall survival (*p* < 0.001). We then established a prognostic nomogram including tumor size, pathologic T, N, M stage, coagulative necrosis, lymphovascular invasion and fucosyltransferase-III expression. Furthermore, the predictive accuracy of the Leibovich prognostic score was improved when fucosyltransferase-III expression was added (*p* = 0.009 for overall survival and *p* = 0.002 for recurrence-free survival).

**Materials and Methods:**

We conducted a retrospective cohort study of 406 patients who underwent partial or radical nephrectomy between January 2008 and December 2009 in a single institute. Fucosyltransferase-III expression levels were evaluated by immunohistochemical staining in tumor tissues. Kaplan-Meier method was applied to compare survival curves. Cox regression models were fitted to analyze the effect of prognostic factors on recurrence-free and overall survival. Harrell’s concordance index and Akaike’s Information Criteria were calculated to assess predictive accuracy.

**Conclusions:**

Fucosyltransferase-III is a predictive factor for poor overall survival and recurrence free survival in patients with ccRCC. The inhibitor of fucosyltransferase-III might be a potential therapeutic method for the disease.

## INTRODUCTION

The incidence of kidney cancer is highest in North America and Western Europe, and increasing quickly in developing country such as China these years [1, 2]. In China, the incidence of kidney cancer was 68.8 per 100,000 and the mortality was 23.4 per 100,000 in 2015. Renal cell carcinoma (RCC) represents about 90 percents of kidney malignancies [2]. RCC is a group of heterogeneous diseases for its diversity of pathologic subtypes which are different in biological behavior and prognosis due to its different pathologic subtype [3]. The RCC patients have a 5-year survival rate for 70% to 90% if their tumor is localized. Nevertheless, the 5-year survival rate was below 10% if the metastasis exists [4, 5]. Clear cell RCC (ccRCC) is the most common pathologic subtype of RCC [3, 6]. It has been elucidated that the deactivation of the von Hippel-Lindau gene, a tumor-suppressor gene, can trigger the formation of ccRCC. However, the theory cannot explain the different biological behavior of ccRCC. Therefore, some biomarkers are needed to predict the prognosis of ccRCC patient more accurately.

Fucosyltransferases (FUTs or Fuc-Ts, FUT-I to FUT-XI) are a family of enzymes catalyzing the reaction of fucosylation. Fucosylation is an important part of post-translational modification found to be associated with tumorigenesis and the malignant potential of the tumor cells [7, 8]. Synthesis of N-linked glycans and O-linked glycans both need the catalysis of the FUTs. Some of the FUTs catalyze the terminal fucosylation, for example, the last step of the synthesis of Sialic-Lewis X(SLe^x^). And some of the FUTs catalyze core fucosylation, such as FUT-VIII is engaged in the addition of α-1,6-fucose to the N-acetylglucosamine (GlcNAc) residue in some molecule in breast cancer and lung cancer [9]. It has been elucidated that the increased core fucosylation of α-fetoprotein can be used to distinguish hepatocellular carcinoma from liver cirrhosis and chronic hepatitis [10]. In breast cancer, the dimerization and phosphorylation of epidermal growth factor receptor (EGFR) is increased if it is over-fucosylated, and this lead to the increase of downstream EGFR-signaling, which promotes the malignant behavior and growth of the tumor [11].

Fucosyltransferase-III (FUT3, FucT-III) is one of the enzymes from FUTs family. FUT3 is coded by *FUT3* gene (also called Lewis gene), which located in the 19p13.3. FUT3 is an enzyme with α(1,3)-fucosyltransferase and α(1,4)-fucosyltransferase activities. The most well-known function of FUT3 is the biosynthesis of the Lewis blood-group antigen. In oncologic researches, FUT3 has been found to be up-regulated in cancerous tissue of human colorectal cancer [12]. Down-regulation of FUT3 and FUT5 by shRNA technique can weaken the capability of adhesion to endothelial cell because of the reduced binding to E-selectin and hyaluronic acid [7]. Some cell receptor, such as Transforming growth factor - beta (TGF-β), can transduce a signal for epithelial-mesenchmal transition (EMT) if the TGF-β is fucosylated under the catalysis of FUT3. In addition, they also find the patients with metastatic colorectal cancer (mCRC) have a high expression of FUT3 [13]. Though there are some studies focused on FUTs and fucosylated glycans these years, the function of FUT3 in tumorigenesis and the correlation between FUT3 and malignancies or ccRCC still remains unclear.

In this study, we sought to uncover the relations between FUT3 expression and the prognosis of the ccRCC patients. Our findings demonstrated the high expression of FUT3 could predict a poor prognosis in patients with ccRCC. The expression of FUT3 can stratify the patients into two groups with significant difference in overall survival (OS) and recurrence free survival (RFS). In addition, we built models to predict the OS and RFS of ccRCC patients. Furthermore, we investigated if the predictive accuracy of the existed models, such as TNM stage, was improved after the incorporation of FUT3 expression.

## RESULTS

### Patient characteristics

**Figure 1:**
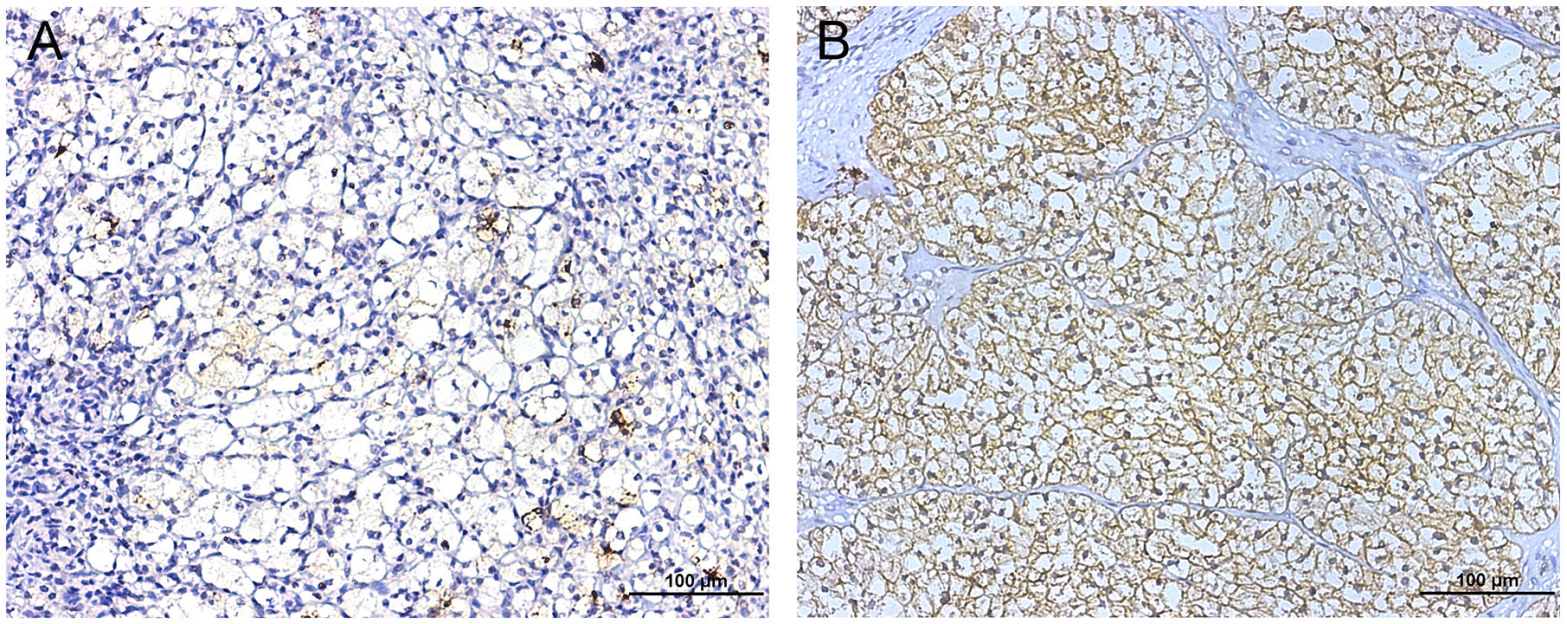
Representative photograph of FUT3 immunostaining in TMA (magnification * 200, scale bar = 100 μm) **(A)** showed low densities of FUT3 while **(B)** showed high densities of FUT3.

To evaluate the level of FUT3 expressed in ccRCC tumor tissues, we conducted the IHC staining to the TMA of 406 patients and analyzed the FUT3 expression of the ccRCC patients. As showed in Table 1 , the mean age of these patients was 55.4 year. The H-score of FUT3 expression ranged from 4 to 220 and representative IHC images were shown in Figure 1. The patients were dichotomized into FUT-3 low group (H-score ranged from 4 to 82; *n* = 230) and FUT-3 high group (H-score ranged from 85 to 220; *n* = 176) according to the method of “minimum *p* value” with the assistance of the X-tile software. The clinical and pathologic features were compared in Table 1 . In general, there was no significant difference of age, gender, tumor size, pathologic N stage, the presence of sarcomatoid change, rhabdoid appearance and LVI between FUT3 high group and FUT3 low group, while pathologic T (*p*=0.006) and M stage (*p*=0.015), Fuhrman grade (*p*=0.005), the presence of coagulative necrosis (*p*=0.022) and ECOG-PS (*p*=0.005) showed a significant difference between two groups.

**Table 1:** Patient characteristics and associations with FUT3 expression

Characteristics	Patients (*n*=406)		FUT3 expression		
	Number	%	Low (*n*=230)	High (*n*=176)	*p* value
Age at surgery:					0.447
Mean ± SD (year)	55.4 ± 12.0		55.1 ± 12.0	55.9 ± 12.1	
Gender:					0.518
Male	285	70.3	158	127	
Female	121	29.7	72	49	
Tumor size:					0.661
Mean ± SD (cm)	4.4 ± 2.5		4.4 ± 2.2	4.5 ± 2.8	
Pathologic T stage:					0.006
pT1	276	68.0	172	104	
pT2	27	6.7	14	13	
pT3	96	23.6	42	54	
pT4	7	1.7	2	5	
Pathologic N stage:					0.094
pN0	399	98.3	229	170	
pN1	7	1.7	1	6	
Pathologic M stage:					0.015
pM0	401	98.8	227	174	
pM1	5	1.2	3	2	
TNM stage:					0.003
I	273	67.2	172	101	
II	25	6.2	12	13	
III	96	23.6	41	55	
IV	12	3.0	5	7	
Fuhrman grade:					0.005
1	66	16.3	43	23	
2	188	46.3	116	72	
3	99	24.4	51	48	
4	53	13.1	20	33	
Coagulative necrosis:					0.022
Absent	316	77.8	189	127	
Present	90	22.2	41	49	
Sarcomatoid change:					0.939
Absent	393	96.8	223	170	
Present	13	3.2	7	6	
Rhabdoid appearance:					0.126
Absent	383	94.3	221	162	
Present	23	5.7	9	14	
LVI:					0.198
Absent	298	73.4	175	123	
Present	108	26.6	55	53	
ECOG-PS:					0.005
= 0	335	82.6	201	134	
> 1	71	17.4	29	42	

Abbreviations: FUT3 = Fucosyltransferase 3; LVI = lymphovascular invasion; ECOG-PS: Eastern Cooperative Oncology Group – Performance status.

### High FUT3 expression predicted poor OS and RFS in patients with ccRCC

At the time of last follow-up, 74 (18.2%) patients had recurrence of RCC, including 19 patients with local recurrence only, 46 patients with distant recurrence only and 9 patients with both local and distant recurrence.

**Figure 2:**
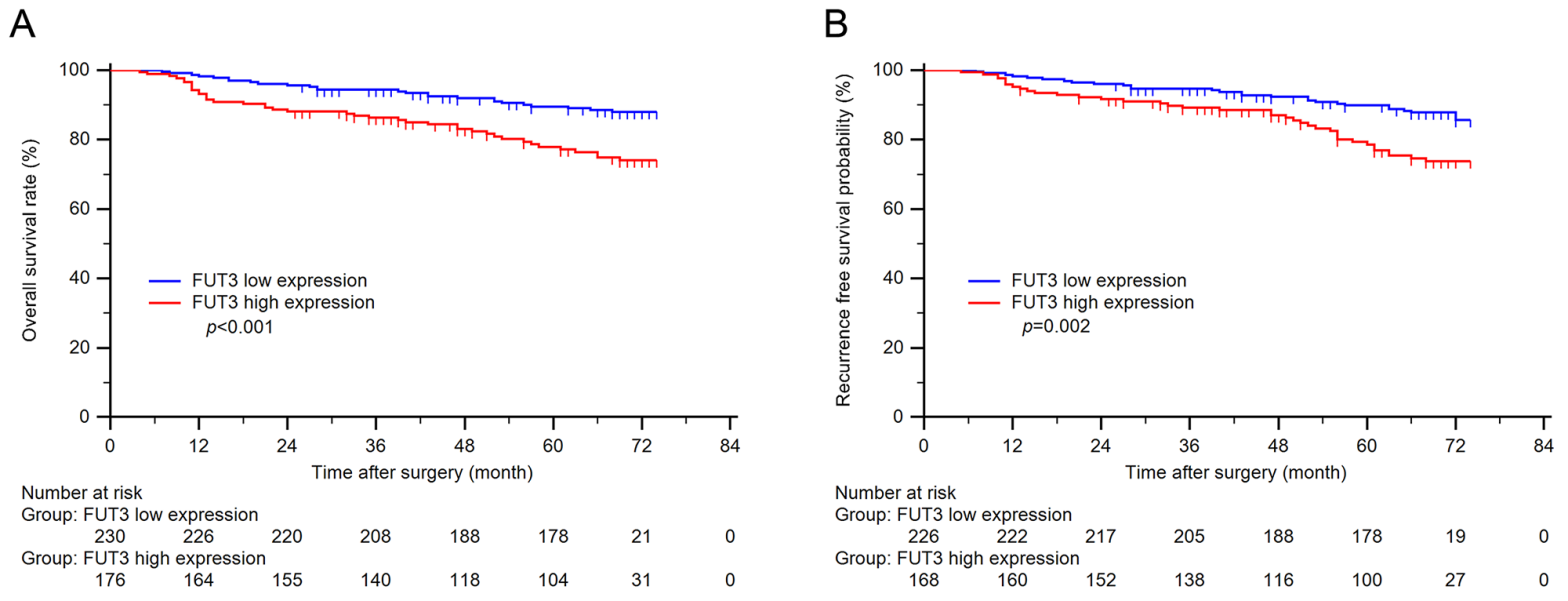
Kaplan-Meier curves of overall survival and recurrence free survival based on tumor FUT3 expression **(A)** n = 406, *p* < 0.001; **(B)** n = 394, p = 0.002.

Kaplan-Meier method and log-rank were conducted to evaluate the relationship between FUT3 expression and clinical outcomes in ccRCC patients. As shown in Figure 2 FUT3 expression significantly correlated with OS (*p* < 0.001) as well as RFS (*p* = 0.002) in patients with ccRCC.

### FUT3 was an independent factor for poor prognosis in ccRCC patients

In order to confirm the prognostic significance of FUT3 expression and other clinicopathologic features in ccRCC, univariate cox analysis was applied. As in Table 2 , in univariate cox regression, tumor size (*p* < 0.001), pathologic T stage (*p* < 0.001), N (*p* < 0.001), M stage (*p* = 0.003), Fuhrman grade 3 and 4 (*p* < 0.001), sarcomatoid change (*p* < 0.001), rhabdoid appearance (*p* < 0.001), coagulative necrosis (*p* < 0.001), lymphovascular invasion (*p* < 0.001), ECOG-PS (*p* < 0.001) and FUT3 expression (*p* < 0.001) had significant impacts on OS.

**Table 2:** Univariate Cox regression analyses of potential prognostic factors for overall survival and recurrence free survival in ccRCC patients

Variable	Overall survival		Recurrence free survival	
	Hazard ratio (95% CI)	*p*	Hazard ratio (95% CI)	*p*
Tumor size:	1.482 (1.368-1.605)	<0.001	1.510 (1.382-1.650)	<0.001
Pathologic T stage:				
pT2 vs. pT1	4.060 (1.660-9.926)	0.002	4.228 (1.597-11.196)	0.004
pT3 vs. pT1	5.219 (3.073-8.864)	<0.001	4.938 (2.775-8.786)	<0.001
pT4 vs. pT1	11.796 (4.537-30.668)	<0.001	13.585 (5.170-35.696)	<0.001
Pathologic N stage:				
pN1 vs. pN0	36.464 (15.261-87.125)	<0.001	/	/
Pathologic M stage:				
pM1 vs. pM0	5.800 (1.815-18.536)	0.003	/	/
Fuhrman grade:				
2 vs. 1	1.562 (0.528-4.616)	0.442	1.387 (0.463-4.155)	0.561
3 vs. 1	4.162 (1.422-12.183)	0.010	3.584 (1.203-10.676)	0.023
4 vs. 1	17.309 (6.049-49.528)	<0.001	15.008 (5.163-43.626)	<0.001
Sarcomatoid change:				
Present vs. Absent	19.680 (9.178-42.196)	<0.001	26.191 (10.910-62.878)	<0.001
Rhabdoid appearance:				
Present vs. Absent	9.308 (5.187-16.704)	<0.001	8.602 (4.419-16.745)	<0.001
Coagulative necrosis:				
Present vs. Absent	4.418 (2.734-7.140)	<0.001	3.967 (2.351-6.693)	<0.001
LVI:				
Present vs. Absent	4.501 (2.779-7.289)	<0.001	4.466 (2.651-7.525)	<0.001
ECOG-PS:				
≥1 vs. 0	5.004 (3.037-8.245)	<0.001	3.992 (2.270-7.020)	<0.001
FUT3 expression:				
High vs. Low	2.342 (1.436-3.821)	<0.001	2.076 (1.230-3.505)	0.006

Abbreviations: ccRCC = clear cell renal cell carcinoma; FUT3 = Fucosyltransferase 3; LVI= lymphovascular invasion; ECOG-PS: Eastern Cooperative Oncology Group – Performance status.

On the other hand, as presented in Table 2 , in univariate cox regression, tumor size (*p* < 0.001), pathologic T stage (*p* < 0.001), Fuhrman grade 3 and 4 (*p* < 0.001), sarcomatoid change (*p* < 0.001), rhabdoid appearance (*p* < 0.001), coagulative necrosis (*p* < 0.001), lymphovascular invasion (*p* < 0.001), ECOG-PS (*p* < 0.001) and FUT3 expression (*p* = 0.006) had a significant impact on RFS.

**Figure 3:**
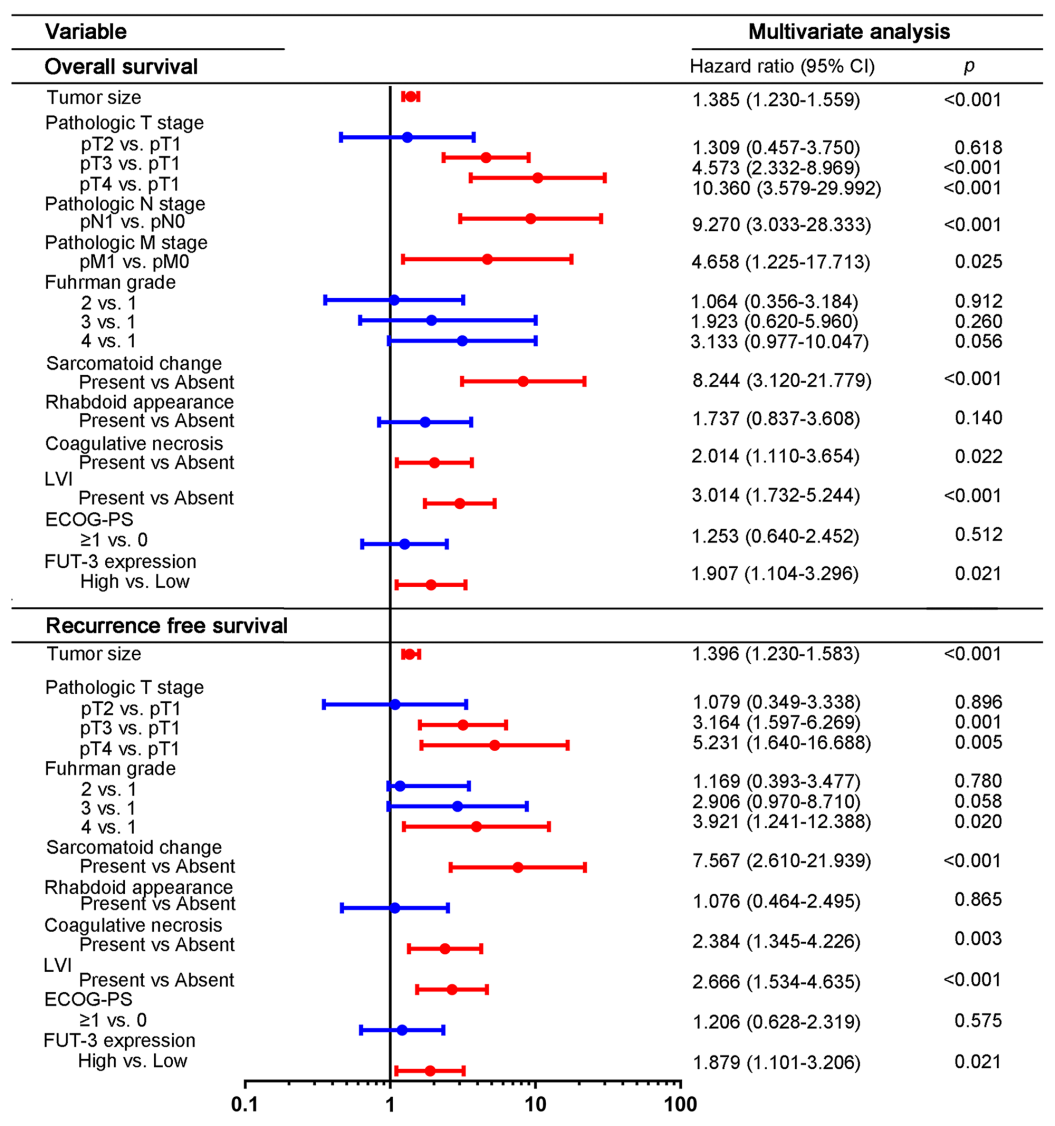
Multivariate analyses of conventional prognostic features in OS and RFS Figure 3 showed hazard ratio and *p*-value of each clinical and pathologic feature for OS and RFS. Abbreviation: LVI, lymphovascular invasion; ECOG-PS, Eastern cooperative Oncology Group performance status; FUT3, fucosyltransferase-III.

Then the multivariate cox analysis was then conducted to the above significant factors. Result in Figure 3 indicated that FUT3 expression was an independent prognostic factor in ccRCC, both for OS (HR = 1.907; *p* = 0.021) and RFS (HR = 1.879; *p* = 0.021). The other factors which considered statistically significant and independent prognostic factors included: pathologic T, N, M stage, presence of sarcomatoid change, coagulative necrosis and lyphovascular invasion for OS and pathologic T stage, Fuhrman grade, presence of sarcomatoid change, coagulative necrosis and lyphovascular invasion for RFS.

### Construction of prognostic nomogram for OS and RFS in patients with ccRCC

**Figure 4:**
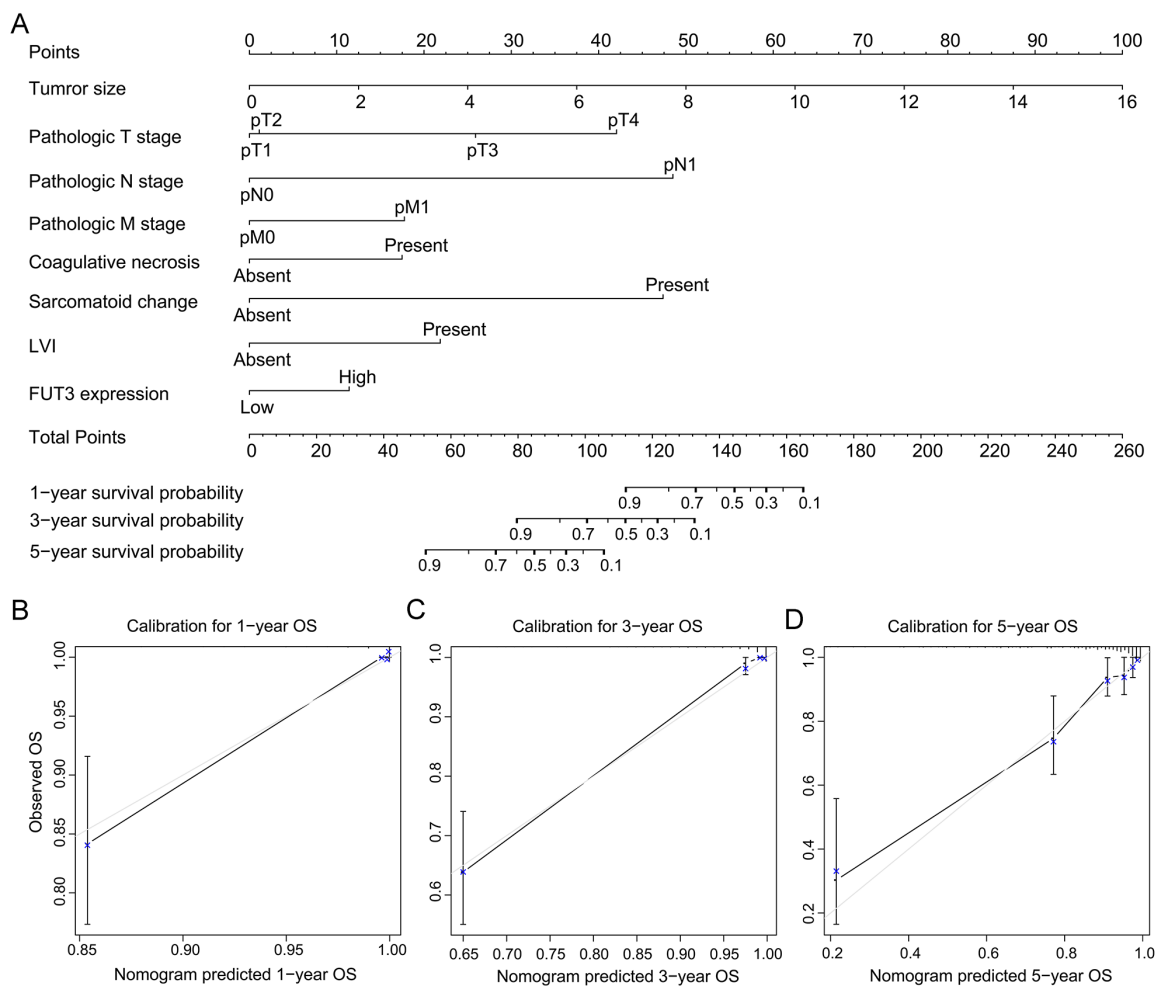
Built-up prognostic nomogram for OS prediction and calibration for it **(A)** showed an eight independent prognostic factors including tumor size, pathologic T, N, M stage, the presence of coagulative necrosis and lymphovascular invasion and FUT3 expression. The total point was generated by adding the scores of the prognostic factors. **(B-D)** showed the calibration plot for nomogram predicted 1-year, 3-year and 5-year overall survival rate.

**Figure 5:**
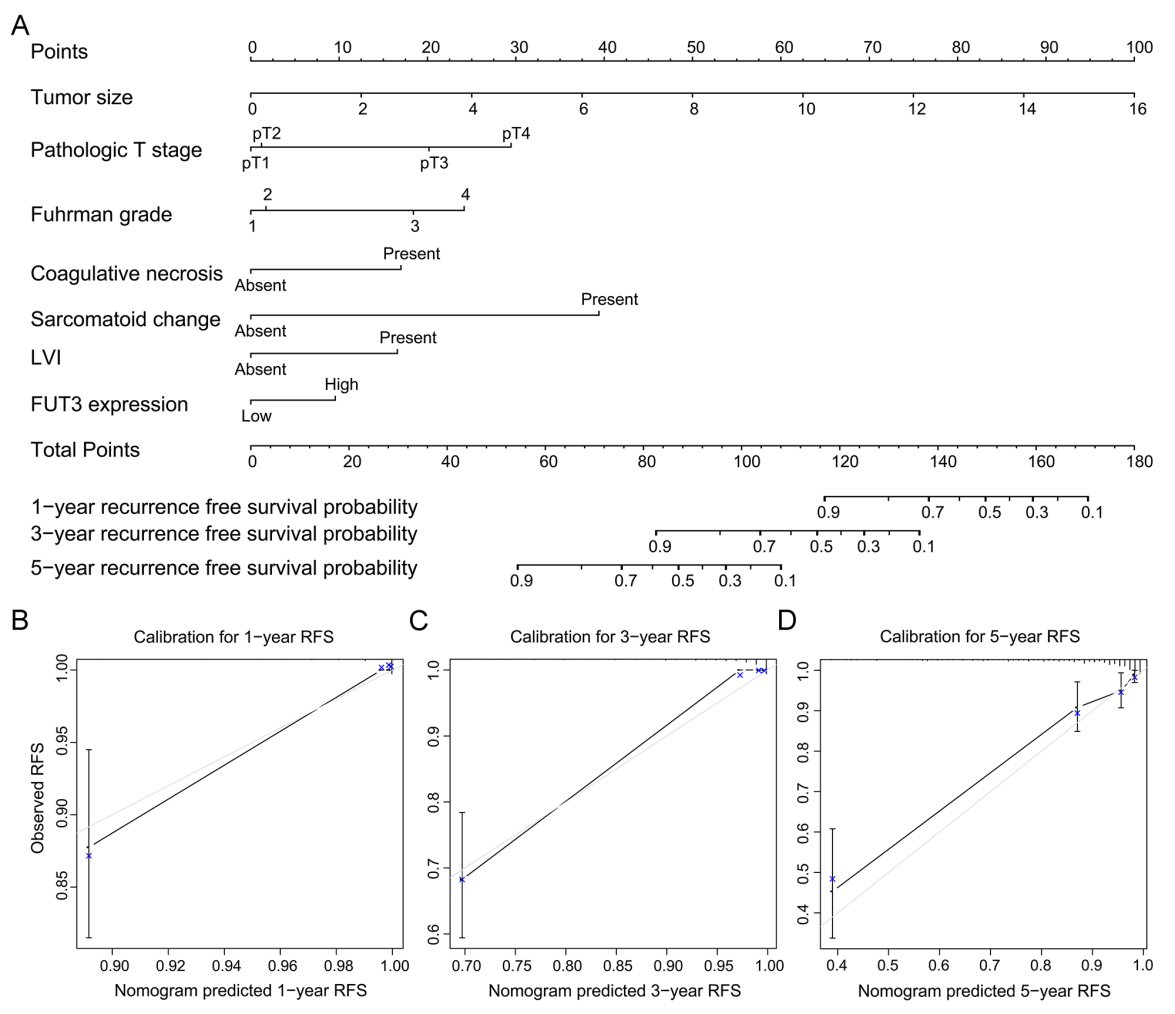
Built-up prognostic nomogram for RFS prediction and calibration for it **(A)** showed a seven independent prognostic factors including tumor size, pathologic T stage, Fuhrman grade, the presence of coagulative necrosis and lymphovascular invasion and FUT3 expression. The total point was generated by adding the scores of the prognostic factors. **(B-D)** showed the calibration plot for nomogram predicted 1-year, 3-year and 5-year recurrence free survival rate.

All these independent prognosticators were brought into the prognostic nomogram of OS (Figure 4A) and RFS (Figure 5A). The calibration plot for the prognostic accuracy of OS and RFS at 1, 3, 5 years after surgery had a good agreement between the predicted and observed survival (Figure 4B-4D, Figure 5B-5D).

### Ability of FUT3 expression to enhance the established prognostic models

As presented above, high FUT3 expression correlated with reduced survival and high probability in patients with ccRCC. We further investigated whether the addition of FUT3 expression to established prognostic models can improve their predictive accuracies. The C-index, AIC and *p* value were presented in Table 3 . For OS, the predictive accuracy of Leibovich model improved from 0.829 to 0.854 (*p* = 0.009). However, the combination of FUT3 expression with TNM grade, SSIGN or UISS did not improve the predictive accuracies of the models. On the other hand, for RFS, the predictive accuracies of TNM grade (*p* = 0.019), UISS (*p* = 0.004), SSIGN (*p* = 0.009) and Leibovich model (*p* = 0.002) were all improved by the addition of FUT3.

**Table 3:** The predictive value of FUT3 and the combined hazard models in ccRCC patients

	Overall survival			Recurrence free survival		
Models	C-index	AIC	*p* value	C-index	AIC	*p* value
FUT3	0.606	773.041		0.608	847.870	
TNM stage	0.726	731.536	0.088	0.683	826.163	0.019
TNM stage + FUT3	0.751	727.496		0.725	819.997	
UISS	0.770	701.346	0.111	0.762	777.415	0.004
UISS + FUT3	0.789	699.420		0.785	774.583	
SSIGN	0.778	679.953	0.042	0.761	760.206	0.009
SSIGN + FUT3	0.807	675.563		0.798	754.550	
Leibovich	0.829	654.813	0.009	0.818	731.214	0.002
Leibovich + FUT3	0.854	652.612		0.849	727.700	

Abbreviations: ccRCC = clear cell renal cell carcinoma; FUT3 = Fucosyltransferase 3; UISS = University of California Los Angeles Integrated Staging System; SSIGN = Stage Size, Grade and Necrosis; *p* value = *p* valuefor AIC.

## DISCUSSION

Our study confirms the significance of FUT-3 expression for patients with ccRCC. The high expression of FUT3 can predict poor prognosis of ccRCC patient. Furthermore, we built nomogram models for OS and RFS of ccRCC patients and confirm the models with calibration. In addition, we combine the built-up models and the expression of FUT-3 to improve the predictive accuracy.

FUT-3 is an enzyme from the FUTs family, and is highly expressed in stomach, colon, intestine, lung, and kidney [9]. *FUT3* gene is also known as Lewis gene, for its significance in biosynthesis of Lewis antigen (Le) and sialic Lewis antigen (sLe). Many studies from other research groups had similar results to our study, in other words, FUT-3 can promote the malignant behavior of the tumor and lead to a poor prognosis. The high expression of FUT-3 and its product, tetrasaccharide Sialic Lewis x (sLe^x^) has been observed in several kinds of malignant solid tumors, such as oral squamous cell carcinoma [14], breast invasive ductal carcinoma [15], pancreatic cancer [16], ovarian carcinoma and colorectal cancer [17]. sLe^x^ is the well-known ligands of the E-selectin [18], one of the cell-adhesion molecules of the endothelium around the blood vessels. E-selectin is a kind of Ca2+-dependent C-type lectins, which mediates cell-cell adhesion in vessels when inflammation takes place [9]. This adhesion is necessary for leukocyte rolling on the endothelium and then escape to inflammatory sites [19]. Nevertheless, this pattern of cell-cell adhesion also allows tumor cell to extravasate to other place during hematogenous metastasis [20], and then promote the metastasis of cancer. A recent study illustrated the proliferation and other malignant properties such as migration and invasion capability of gastric carcinoma cell line can be inhibited by *FUT3* gene silencing [21]. In contrast, we cannot confirm that the sLe^x^ is all synthesized by FUT3, since FUT4, FUT5, FUT6, FUT-9 and FUT7 also have the α(1,3)-fucosyltransferase activity and their function in Lewis antigen synthesis of different tissue has as well been proved [22–24].

Another well-known fucosylated glycan, sialic Lewis a (sLe^a^, CA19-9), is synthesized specifically by FUT3, because FUT3 has the only α (1,4)-fucosyltransferase activity in the FUTs family. sLea is another ligand for E-selectin and a meaningful circulatory biomarker of pancreatic carcinoma and some colorectal cancer in prognosis prediction and recurrence surveillance [9]. Nevertheless, the significance of sLe^a^ in prognosis prediction and recurrence surveillance of ccRCC patients and how it participates in the tumorigenesis and malignant transforming still remains unknown.

Furthermore, CD47 as a glycoprotein on the cellular membrane can give an inhibitory signal to the macrophage by binding to the signal regulatory protein alpha (SIRPα) on the surface of macrophage [25]. Fucosylated glycans, Lewis Y (Le^y^), can enhance the capacity of CD47-SIRP signaling and then enables the tumor cell to evade the phagocytosis or programmed cell clearance [26, 27].

Some studies demonstrated the opposite results to the studies mentioned above. Low FUT3 mRNA is correlated with the migration and liver metastasis of the colorectal cancer, which means FUT3 is a factor for poor prognosis of patients with colorectal cancer [28]. This paradox may be a result of tumor micro-environment. NK cell can recognize the sLex expressed on the tumor-cell surface and trigger the cytotoxicity mediated by CD94 and NKG2D [29]. On the other hand, the extrinsic apoptosis pathway is mediated by fucosylation in the TNF-related apoptosis-inducing ligand (TRAIL). The pathway is activated when TRAIL is combined to the TRAIL receptors, and then leads to the apoptosis of the target cells [30]. The FUT3 expression upregulates sensitivity of TRAIL pathway in colon cancer patients [31].

A few shortcomings of this study should be noted. The major limitation of this study is the relatively small sample size of the cohort and the retrospective design. A multi-center prospective study in cohorts with larger sample size is needed to confirm these results. TMA technique only displays small representative part of the tumor, and expression of FUT3 is subjectively evaluated.

## CONCLUSION

In summary, FUT3 was a predictive factor for poor OS and RFS in patients with ccRCC. FUT3 enhanced the capability of established RCC prognostic models. Inhibitor of FUT3 might be a potential therapeutic method for ccRCC. The FUT3 in blood or tumor tissue would be new biomarkers for ccRCC detection or prognosis prediction soon.

## MATERIALS AND METHODS

### Patients

The cohort of this retrospective study included 406 ccRCC patients from Department of Urology, Zhongshan Hospital, Fudan University. The inclusion criteria were (1) ccRCC proved by histopathology; (2) underwent a radical nephrectomy or a partial nephrectomy between January, 2008 and December, 2009; (3) no history of another malignant tumor. For each patient, we collected the age at surgery, gender, Eastern cooperative Oncology Group performance status (ECOG-PS), TNM stage [32], Fuhrman grade [33], tumor size, and pathologic features of the tumor, such as coagulative necrosis, rhabdoid appearance, sarcomatoid change, lymphovascular invasion (LVI). Coagulative necrosis, rhabdoid appearance and sarcomatoid change was defined as these pathologic changes observed under the microscope. LVI was defined as the appearance of tumor cells in a space lined with endothelium without underlying muscular walls. SSIGN score, UISS score and Leibovich score were applied to the patients [34–36].

Patients underwent a nephrectomy if their disease was localized while patients with metastatic disease received interferon-α-based immunotherapy after cytoreductive nephrectomy. Patients were followed up with physical examination, laboratory tests, chest X-ray and abdominal CT scan or ultrasound postoperatively every half year for the first two years and annually from then on. The follow-up data was lastly updated March 2015. The study was approved by the clinical research ethic committee of Zhongshan Hospital, Fudan University and informed consent was signed and obtained from each patient.

### Immunohistochemistry and assessment

We performed the immunohistochemical (IHC) staining on the tissue microarray (TMA). In the TMA construction, we chose the area away from hemorrhage and necrosis as the representative area. Rabbit polyclonal anti-FUT3 antibody (1:50 dilution, ab110082, Abcam, Cambridge, MA) was used for IHC staining. The IHC score was assessed by two independent pathologists, who were blind to the clinical or pathologic data. A semi-quantitative H-score was generated by multiplying the staining intensities (0 as negative, 1 for weak, 2 for moderate, and 3 for strong staining intensity) by the percentages of positive staining area. So the H-score of each sample ranged from 0 to 300.

### Statistical analysis

X-tile plot analysis was applied to select the suitable cutoff value of the H-score according to the “minimum *p* value” method, in order to set the patients apart into two subgroups of low and high expression of FUT3 [37]. We evaluated the comparison between the expression of FUT3 and clinical and pathological variables by using the student’s t test, χ ^2^ test and Wilcoxon rank-sum test. OS curves and RFS curves were generated by Kaplan-Meier method and compared by log-rank t test. Independent associations between OS or RFS and clinicopathologic variables were evaluated by using univariable and multivariable Cox proportional-hazard models. Nomogram was built to predict the prognosis, and its prediction accuracy is evaluated by calibration plot. The predictive accuracy and sufficiency of the models were assessed by Harrell’s Concordance index (C-index) and Akaike’s Information Criteria (AIC). The difference between the C-index was compared by Hanley-McNeil test. The difference was considered significant if the two-side *p* value was less than 0.05.

Data were saved and analyzed by using software as followed: X-tile software version 3.6.1, (Yale University, New Haven, CT, USA), GraphPad Prism version 6.02 (GraphPad Software. Inc.), Stata SE, version 12.1 (Stata, College Station, TX), MedCalc Software 11.4.2.0 (MedCalc, Mariakerke, Belgium) and R software 3.1.2 with the “rms” package (R Foundation for Statistical Computing, Vienna, Austria).
